# Cricetidae (Rodentia, Mammalia) from the Oligocene of the Valley of Lakes (Mongolia): the genera *Aralocricetodon*, *Eocricetodon*, *Bagacricetodon*, *Witenia* and *Paracricetodon*

**DOI:** 10.1007/s12549-016-0266-8

**Published:** 2017-02-09

**Authors:** Paloma López-Guerrero, Olivier Maridet, Gudrun Daxner-Höck

**Affiliations:** 1 Geologisch und paläontologische Abteilung, Naturhistorisches Museum Wien, Burgring 7 A, 1010 Vienna, Austria; 2 Collection Management Center, Jurassica Museum, Route de Fontenais 21, 2900 Porrentruy, Switzerland; 3 Department of Geosciences, Earth Sciences, University of Fribourg, Chemin du Musée 6/Pérolles, 1700 Fribourg, Switzerland

**Keywords:** *Aralocricetodon*, *Bagacricetodon*, *Eocricetodon*, Mongolia, Oligocene

## Abstract

We describe the remains of *Aralocricetodon* Bendukidze, 1993; *Bagacricetodon* Gomes Rodrigues et al., 2012; *Eocricetodon* Wang, 2007; *Witenia* de Bruijn et al., 2003 and *Paracricetodon* Schaub, 1925 (Cricetidae, Rodentia, Mammalia) from the Taatsiin Gol and Taatsiin Tsagaan Nuur areas (Mongolia). The studied material (comprising 128 teeth) stems from 9 localities and 23 fossil layers spanning a time interval from ∼33 to ∼24 Ma (early to late Oligocene) and covering the biozones from A to C1. The general dental pattern between the species from the early and late Oligocene differed. The occlusal pattern of the molars was more complicated and the crowns were lower during the early versus late Oligocene. This indicates a change in diet towards more abrasive plants. Several of the studied species were common in both Europe and Asia Minor during the early Oligocene. The species collected from the late Oligocene have also been recorded in Kazakhstan and China. This indicates an interesting biogeographical pattern that merits future study.

## Introduction

The present work presents results on five species of cricetids found in the Oligocene of the Valley of Lakes (Mongolia). Central Asia is one of the centres of mid-Cenozoic mammalian evolution, and its faunas are therefore of special interest for the study of cricetid rodent evolution. This region provides abundant and well-constrained Oligocene fossil sites. The best records of rodents from the Oligocene of Asia stem from localities in Kazakhstan and China (Wang and Meng 1986; Tong 1992, 1997; Wang and Dawson 1994; Emry et al. 1998; Bendukidze 1993; Wang 2007; Lopatin 2004). Recently, the interest in the Oligocene cricetids of Asia has increased substantially (Gomes Rodrigues et al. 2009; Maridet et al. 2009, 2013; Daxner-Höck 2000; Maridet and Ni 2013; Lindsay et al. 2016).

Five genera have been reported in the studied localities: *Aralocricetodon* Bendukidze, 1993; *Bagacricetodon* Gomes Rodrigues et al., 2012; *Eocricetodon* Wang, 2007; *Witenia* de Bruijn et al., 2003 and *Paracricetodon* Schaub, 1925. *Aralocricetodon* was described based on a single M1 found in the deposits of Altynshokysu (Kazakhstan) in 1993. More fossil material was described from this region by Lopatin (1996, 2004). More recently, Bendukidze et al. (2009) revised the material from the localities in the Aral Formation—Altynschokysu (levels 1–4), Sayaken, Akespe and Akotau—offering new descriptions of the type material. *Bagacricetodon* has been recently described at the Ulantatal section (lower upper Oligocene) from Inner Mongolia, China. It is not found elsewhere. *Eocricetodon* was described on the late Eocene from the Houldjin Formation on the Nei Mongol (China) and is considered to be one of the most advanced cricetids from the Eocene. It likely belonged to the basal cricetid stock for Oligocene radiations (Gomes Rodrigues et al. 2012). *Witenia* is a genus from Asia Minor, specifically at the Süngülü A fossil site dated to the Eo/Oligocene boundary interval (de Bruijn et al. 2003). It was recently recorded in the upper Oligocene deposits of the Ulantatal section in Inner Mongolia (Gomes Rodrigues et al. 2012). *Paracricetodon* was described by Schaub in 1925 and it is present during the Oligocene and at the Eo/Oligocene boundary. Several emended diagnoses have been made, but the most recent was published by de Bruijn et al. (2003) and it is also the most complete.

The present contribution is the first citation of some of these genera in Mongolia. *Aralocricetodon*, *Bagacricetodon* and *Eocricetodon* were already documented in the Valley of Lakes (Daxner-Höck et al. 2010; Maridet et al. 2014), but detailed descriptions of the material were lacking until the present paper. *Witenia* and *Paracricetodon* are rare genera described in Asia Minor and mainly found in the European part of Turkey (de Bruijn et al. 2003). We present the revision of these genera from the Taatsiin Gol and Taatsiin Tsagaan areas in Mongolia (for geological details, see Daxner-Höck et al. 2017, this issue). Here we provide a detailed description of the species found in the early and late Oligocene, sumplementing all the previous works made on the Mongolian cricetids (Daxner-Höck et al. 2010; Maridet et al. 2014; López-Guerrero et al. 2015, 2016, 2017, this issue).

## Material and methods

### Institutional abbreviations

*NHMW*: Naturhistorisches Museum Wien, Vienna, Austria.

### Locality abbreviations

*TAT*: Tatal Gol; *TGR*: Taatsin Gol Right; *TGL*: Taatsin Gol Left; *SHG*: Hsanda Gol; *DEL*: Del; *IKH*: Ikh Argalatyn Nuruu; *UNCH*: Unkheltseg; *ABO*: Abzag Ovo; *TAR*: Unzing Churum; *TGW*: Torglorhoi.

### Material

The studied material includes 128 upper and lower molars from 9 localities (23 fossil layers) of Oligocene age. They belong to five species of *Aralocricetodon*, *Bagacricetodon*, *Eocricetodon*, *Witenia* and *Paracricetodon*. These fossils are stored in the collections of the Geologisch-Paläontologische Abteilung, Naturhistorisches Museum Wien (Austria). Table 1 shows the number of molars examined from each fossil layer and the biochronological information of the localities (for more details, see Daxner-Höck et al. 2017, this issue and Harzhauser et al. 2017, this issue). The sites belong to the local biozones A and B from the early Oligocene and to C and C1 from the late Oligocene (Daxner-Höck et al. 2010). They can be correlated to the Rupelian (A–B) and Chattian (C–C1) stages. We have compared the Mongolian material with the collection from the Ulantatal section (China) stored at the Institute of Vertebrate Paleontology and Paleoantropology in Beijing (China) and with the casts of the material from Altynshokysu (Bone Bed 2) from Kazakhstan stored at the NHMW. The terminology used to describe the teeth is adapted from Maridet et al. (2009), Maridet and Ni (2013) and López-Guerrero et al. (2017, this issue); nevertheless, we use the terms metalophulid I and II for protoconid hind arm and metalophulid, respectively. Anatomical abbreviations for upper molars are M1, M2 and M3, and for lower molars m1, m2 and m3. Observations and measurements were carried out using a binocular microscope Zeiss Discovery V20. Maximum length and width measurements for each specimen, given in millimetres, were taken using Carl Zeiss software Axiocam MRc5 by means of a digital camera attached to a microscope. All the measurements are given in Table 2. Photographs were taken with a Philips XL 30 scanning electron microscope at the Core Facility of Cell Imaging and Ultrastructure Research (CIUS), EM LAB, Faculty of Life Sciences, University of Vienna (Austria). We have followed the classifications of Mein and Freudenthal (1971) and Wilson and Reeder (2005), which recognised the status of the family Cricetidae.

**Table 1:** Studied material from the Taatsiin Gol and Taatsiin Tsagaan Nuur areas

Biozone	Species	Locality	M1	M2	M3	m1	m2	m3	Total
C1	*A. schokensis*	TAT-E/surf	1	1					**2**
C1	*A. schokensis*	IKH-A/5					1		**1**
C1	*A. schokensis*	DEL-B/12			1	1		1	**3**
C	*A. schokensis*	TGW-A/2b					2		**2**
C	*A. schokensis*	TGW-A/2a			1	1		1	**3**
C	*A. schokensis*	TAR-A/2	3	2		1			**6**
C	*A. schokensis*	TGR-C/2	3	1	2	2	3	2	**13**
C	*A. schokensis*	TGR-C/1	5	2		5	8	4	**24**
		**Total**	**12**	**6**	**4**	**10**	**14**	**8**	**54**
C1	*B. tongi*	DEL-B/12				3			**3**
C	*B. tongi*	TGW-A/2b	3	3	1	10	8	1	**26**
C	*B. tongi*/*B.* cf. *tongi*	TGW-A/2a	1		1				**2**
C	*B. tongi*	ABO-083				2	1		**3**
C	*B. tongi/B.* cf. *tongi*	TGR-C/1	2	1	1	1			**5**
		**Total**	**6**	**4**	**3**	**16**	**9**	**1**	**39**
C1	*E. meridionalis*	TAT-E/27	1						**1**
C	*E. meridionalis*	ABO-A/3			1				**1**
C	*E. meridionalis*/*E.* cf. *meridionalis*	TGW-A/2a				5	3	1	**9**
C	*E. meridionalis*	TGR-C/5				1			**1**
B	*E. meridionalis*	UNCH-A/3B	1						**1**
B	*E. meridionalis*	TGR-AB/22				1	1	1	**3**
B	*E. meridionalis*/*E.* cf. *meridionalis*	TGR-ZO/2				1	1	2	**4**
B	*E. meridionalis*	TGR-B/1				2	1		**3**
B	*E. meridionalis*	IKH-A/1				1			**1**
B	*E. meridionalis*	DEL-B/7		1	1	1	1		**4**
A	*E. meridionalis*	TGR-A/13	1						**1**
A	*E. meridionalis*	SHG-AB/17-18				2			**2**
A	*E. meridionalis*	SHG-C/1		1		1			**2**
		**Total**	**3**	**2**	**2**	**15**	**7**	**4**	**33**
A	*Paracricetodon*	TGR-A/14		1					**1**
B	*Witenia*	UNCH-A/3		1					**1**

Local biozones after Daxner-Höck et al. (2010) (A and B early Oligocene; C and C1 late Oligocene). For the localities abbreviations, see Daxner-Höck et al. (2017, this issue)

**Table 2:** Lengths and widths of the upper and lower molars taken of the studied species from the Valley of Lakes (Mongolia)

		Length				Width				*L*/*W*
		*N*	Min	Mean	Max	*N*	Min	Mean	Max	
*A. schokensis*										
M1	TAT-E/surf	1	–	2.46	–	1	–	1.61	–	1.528
	TAR-A/2	2	2.14	2.15	2.18	2	1.56	1.59	1.62	1.352
	TGR-C/2	2	1.98	2.11	2.25	3	1.45	1.54	1.67	1.370
	TGR-C/1	5	2.07	2.16	2.32	5	1.45	1.62	1.80	1.334
M2	TAT-E/surf	1	–	1.86	–	1		1.53	–	1.216
	TAR-A/2	2	1.71	1.76	1.82	2	1.60	1.62	1.64	1.086
	TGR-C/2	1	–	1.61	–	1	–	1.62	–	0.994
	TGR-C/1	1	–	1.73	–	1	–	1.52	–	1.138
M3	DEL-B/12	1	–	1.40	–	1	–	1.37	–	1.022
	TGW-A/2a	1	–	1.43	–	1	–	1.52	–	0.941
	TGR-C/2	1	–	1.50	–	1	–	1.50	–	1.000
m1	TGW-A/2b	1	–	2.00	–	1	–	1.33	–	1.504
	TGR-C/2	2	1.79	1.85	1.88	2	1.34	1.38	1.41	1.341
	TGR-C/1	4	1.81	1.88	1.98	4	1.24	1.38	1.42	1.362
m2	IKH-A/5	1	–	1.82	–	1	–	1.40	–	1.300
	TGW-A/2b	1	–	1.84	–	1	–	1.33	–	1.383
	TGW-A/2a	1	–	1.84	–	1	–	1.38	–	1.333
	TAR-A/2	1	–	1.91	–	1	–	1.54	–	1.240
	TGR-C/2	3	1.82	1.91	2.05	3	1.54	1.56	1.58	1.224
	TGR-C/1	7	1.81	1.89	2.05	7	1.40	1.49	1.58	1.268
m3	DEL-B/12	1	–	1.77	–	1	–	1.54	–	1.149
	TGW-A/2a	1	–	1.66	–	1	–	1.51	–	1.099
	TGR-C/2	2	1.76	1.97	1.98	2	1.43	1.44	1.46	1.368
	TGR-C/1	3	1.63	1.66	1.70	4	1.40	1.41	1.44	1.117
*B. tongi*/*B*. cf. *tongi*										
M1	TGW-A/2b	3	1.86	2.03	2.17	3	1.20	1.32	1.39	1.538
	TGW-A/2a	1	–	1.89	–	1	–	1.17	–	1.618
	TGR-C/1	2	2.00	2.09	2.17	2	1.48	1.51	1.53	1.384
M2	TGW-A/2b	3	1.38	1.49	1.58	3	1.17	1.27	1.32	1.173
	TGR-C/1	1	–	1.70	–	1	–	1.50	–	1.134
M3	TGW-A/2b	1	–	1.09	–	1	–	1.10	–	0.993
	TGW-A/2a	1	–	1.28	–	1	–	1.24	–	1.027
	TGR-C/1	1	–	1.20	–	1	–	1.23	–	0.975
m1	DEL-B/12	1	–	1.63	–	2	1.00	1.01	1.02	1.620
	TGW-A/2b	9	1.55	1.68	1.84	9	0.91	1.03	1.15	1.627
m2	DEL-B/12	1	–	1.48	–	1	–	1.11	–	1.334
	TGW-A/2b	8	1.46	1.53	1.58	8	1.12	1.19	1.22	1.287
m3	TGW-A/2b	1	–	1.25	–	1	–	1.07	–	1.167
	ABO-083	1	–	1.22	–	1	–	1.02	–	1.192
*E. meridionalis*/*E.* cf. *meridionalis*										
M1	TAT-E/27	0	–	–	–	1	–	1.20	–	–
	UNCH-A/3	1	–	1.87	–	1	–	1.35	–	1.385
	TGR-A/13	0	–	–	–	1	–	1.14	–	–
M2	DEL-B/7	1	–	1.38	–	1	–	1.18	–	1.169
	SHG-C/1	1	–	1.36	–	1	–	1.24	–	1.097
M3	DEL-B/7	1	–	0.97	–	1	–	0.98	–	0.990
	ABO-A/3	1	–	0.90	–	1	–	0.83	–	1.084
m1	DEL-B/7	0	–	–	–	1	–	1.04	–	–
	IKH-A/1	1	–	1.46	–	1	–	1.05	–	1.390
	SHG-AB/17-18	2	1.42	1.43	1.44	2	0.95	0.97	0.98	1.474
	TGR-AB/22	1	–	1.33	–	1	–	0.93	–	1.430
	TGR-B/1	1	–	1.48	–	1	–	1.03	–	1.437
	TGR-ZO/2	1	–	1.24	–	1	–	0.90	–	1.378
	TGW-A/2a	1	–	1.44	–	3	0.95	0.99	1.02	1.441
m2	TGW-A/2a	3	1.32	1.33	1.35	3	1.00	1.03	1.05	1.290
	TGR-AB/22	0	–	–	–	1	–	0.90	–	–
	TGR-ZO/2	1	–	1.20	–	1	–	0.98	–	1.224
m3	TGW-A/2a	1	–	1.06	–	1	–	0.93	–	1.140
	TGR-AB/22	1	–	1.33	–	1	–	1.09	–	1.220
	TGR-ZO/2	2	1.11	1.14	1.17	2	0.88	0.93	0.99	1.227

Measurements are in millimetres

*Min* minimum value, *Max* maximum value, *N* number of specimens

## Systematic palaeontology

Order Rodentia Bowdich, 1821

Superfamily Muroidea Illiger, 1811

Family Cricetidae Brandt, 1855

Genus *Aralocricetodon* Bendukidze, 1993

*Aralocricetodon schokensis* Bendukidze, 1993

Fig. 1

**Fig. 1:**
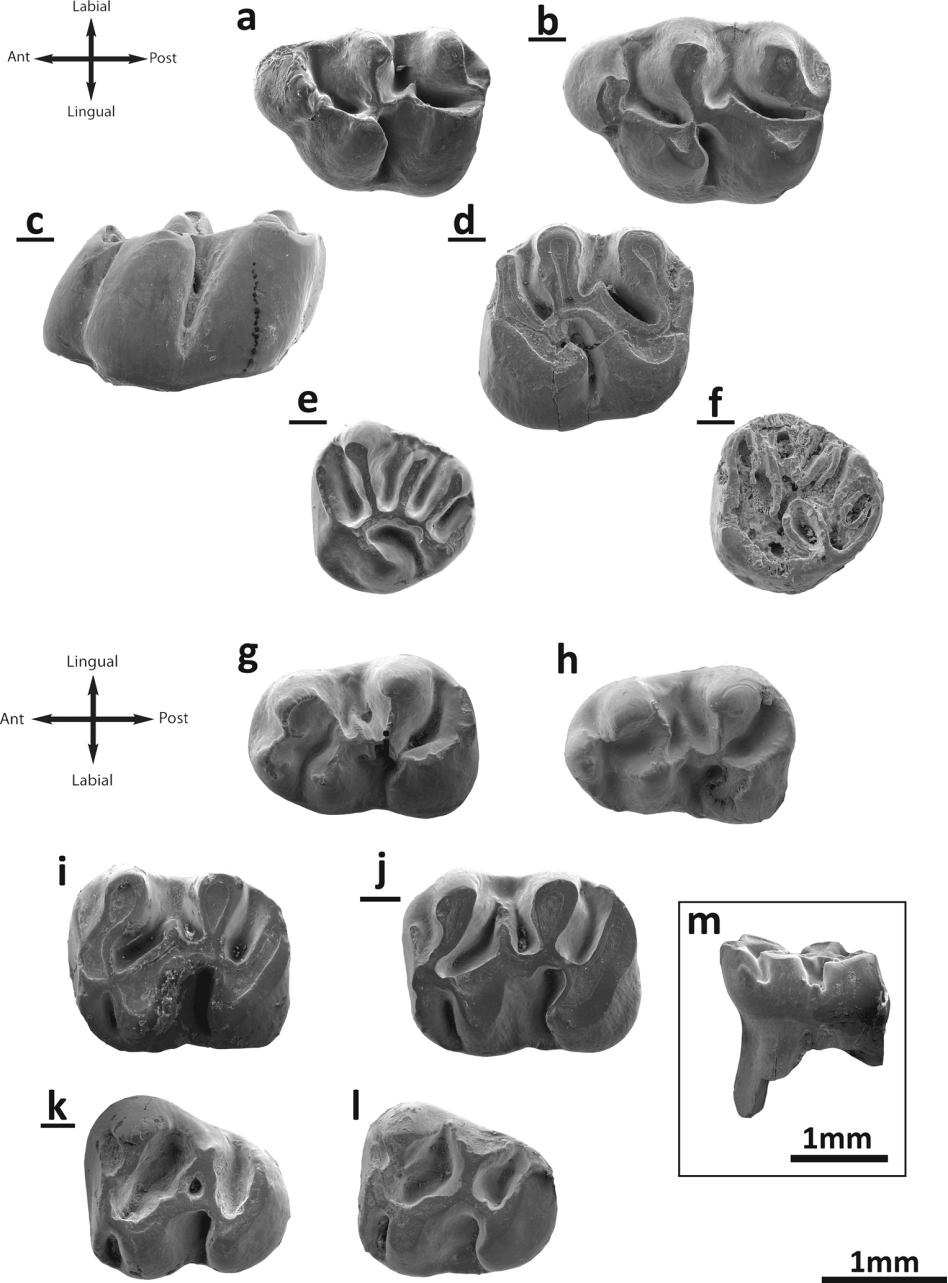
*Aralocricetodon schokensis* from the Valley of Lakes. **a** Taatsiin Gol Right locality, fossil layer TGR-C/2, left M1 (NHMW2015/0322/0003). **b** Fossil layer TGR-C/1, inverted right M1 (NHMW2009z0142/0005). **c** Side view M1 (NHMW2009z0142/0001). **d** Unzing Khurem locality, fossil layer TAR-A/2, inverted right M2 (NHMW2015/0321/0004). **e** Toglorhoi locality, fossil layer TGW-A/2a, inverted right M3 (NHMW2015/0323/0001). **f** Taatsiin Gol Right locality, fossil layer TGR-C/2, inverted right M3 (NHMW2015/0322/0005). **g** TGR-C/1, left m1 (NHMW2009z0142/0014). **h** Toglorhoi locality, fossil layer TGW-A/2a, left m1 (NHMW2015/0323/0002). **i** Unzing Khurem locality, fossil layer TAR-A/2, left m2 (NHMW2015/0321/0007). **j** Taatsiin Gol Right locality, fossil layer TGR-C/2, inverted right m2 (NHMW2015/0322/0009). **k** Del locality, fossil layer DEL-B/12, inverted right m3 (NHMW2015/0325/0002). **l** Taatsiin Gol Right locality, fossil layer TGR-C/1, left m3 (NHMW2009z0142/0019). **m** side view m1 (NHMW2009z0142/0013)

### Synonymy

2014 cf. *Aralocricetodon* sp. Maridet et al. table 3. p. 264

2014 *Aralocricetodon* aff. *schockensis* Maridet et al. table 3. p. 264

2014 *Aralocricetodon* sp. Maridet et al. table 3. p. 264

**Original type locality**: Altynshokysu, North Aral Region, Aral Formation Kazakhstan (lower Oligocene).

**Stratigraphical range**: Oligocene (Mongolia—upper Oligocene: local biozones C and C1), for details see figs. 30–31 in Daxner-Höck et al. (2017) (this issue).

**Geographical range**: Central Asia.

**Material**: see Table 1. Taatsiin Gol Right locality, fossil layer TGR-C/1; catalogue numbers (CN) NHMW2009z0142/0001-19; TGR-C/2 CN: NHMW2015/0322/0001-9. Unzing Khurem locality, fossil layer TAR-A/2; CN: NHMW2015/0321/0001-7. Toglorhoi locality, fossil layer TGW-A/2a; CN: NHMW2015/0323/0001-0003; TGW-A/2b; CN: NHMW2015/0541/0001-2. Ikh Argalatyn Nuruu locality, fossil layer IKH-A/5; CN: NHMW2015/0324/0001. Del locality, fossil layer; CN: NHMW2015/0325/0001-3. Tatal Gol locality, fossil layer TAT-E/surf; CN: NHMW2015/0011/0001-2.

**Measurements**: given in Table 2.

### Description

**M1**: Molar crown relatively high and displaying flat wear (Fig. 1c). Three roots are present, two on the labial part and a wider one on the lingual side. The valleys are deep and narrow. The anterocone is large and transversally elongated at its base but rather narrow at its top. The anterocone is undivided in many specimens, but in those without wear, it can be slightly split at its top (Fig. 1a). A small spur is present in the middle of the anterocone and extends towards the paracone (Fig. 1b); it is absent in some specimens (in layer TGR-C2). The labial anteroloph is present in some molars (TGR-C1; TAT-surf) and reaches the paracone. A narrow anterolophule is always present; it is connected to the lingual part of the anterocone. It is doubled, and in two specimens (TGR-C1), it connects also to a small spur attached to the anterocone. The distal protolophule is always present and connected to the anteroloph (Fig. 1a, b). The anterior arm of the protocone is weak and barely distinguished (Fig. 1b). The paracone is always rounded at its base; it has a short posterior spur in some cases (TAT-surf; Fig. 1a). The mesoloph is present in almost all cases but poorly developed, short or incipient (Fig. 1a, b). The metalophule is posteriorly directed and connected to the posteroloph. The posteroloph is well developed, long and reaches the metacone. The protosinus is poorly developed (Fig. 1a, b). The anterosinus and mesosinus are closed in all specimens by cingula. The sinus is proverse (Fig. 1a, b).

**M2**: The specimens have a well-developed labial anteroloph. A small constriction on the lingual wall of the protocone is present in one specimen; this constriction can be interpreted as an incipient lingual anteroloph (TAT-E/27). The distal protolophule is present and connected to the entoloph (Fig. 1d). The paracone has a posterior spur in some cases (TAT-E/27). The mesoloph is present and is short. Entomesoloph is absent. The metalophule is posteriorly directed and connected to the posteroloph, which is well developed and long. The anterosinus and mesosinus are closed by cingula. The sinus is strongly proverse and closed by a small lingual cingulum.

**M3**: It has rounded outline with a small but distinguished hypocone. The labial anteroloph is present and long and the lingual one is absent or vestigial (Fig. 1e). The mesial protolophule is absent and the anterosinus is deep and narrow. The distal protolophule is long, thin and connected to the anterior part of the entoloph. The entoloph is longitudinally oriented and is connected to the anterior arm of the protocone (Fig. 1e). The mesoloph is always present, located in the middle part of the entoloph (Fig. 1e); it is long and reaches the labial margin. The posterior part of the entoloph is oblique and connected to the anterior arm of the hypocone. The metalophule is long, straight and connected to the point where the anterior arm of the hypocone and the entoloph are joined. The posteroloph is well developed and long. The posterosinus is narrow, and the anterosinus and mesosinus are closed by cingula. The open sinus is long and proverse.

**m1**: The labial anterolophid is usually absent; it is only present in three specimens (TGR-C/1; TGR-C/2; TGW-A/2a; Fig. 1g, h). The lingual anterolophid is absent (Fig. 1g). The anterolophulid is generally absent (Fig. 1g, h), being present in only two specimens (TGR-C/1; TGR-C/2) and connected to the labial part of the anteroconid. It is not present on other teeth (Fig. 1g, h). Both metalophulids, I and II, are always present (Fig. 1g, h). The metalophulid I is connected to the lingual part of the anteroconid. The metalophulid II is long, curved and connected to the protoconid (Fig. 1h) or to the posterior arm of the protoconid (TGR-C/1; TGR-C/2; Fig. 1g). The ectolophid is connected to the middle of the protoconid hind arm (Fig. 1g, h). The anterosinusid is weak and wide. Ectolophid bears a mesolophid. This mesolophid is usually well developed but short (Fig. 1g, h). The ectomesolophid is present in some specimens (Fig. 1g; TGR-C/1), but it is short or incipient. The posterior part of the ectolophid is connected to the anterior arm of the hypoconid. The hypolophulid is short and connected to the ectolophid. The hypoconid hind arm is absent. The posterosinusid is deep and narrow, and the posterolophid displays a constriction. The mesosinusid is closed by a small cingulum. The sinusid is short and wide, transversally directed; it can be closed by a cingulum (Fig. 1h) or by a small stylid (TGR-C/1).

**m2**: Both labial and lingual anterolophids are present, but the lingual one is much less developed (Fig. 1i, j). The anterolophulid is absent. The metalophulid I is present and connected to the anterior arm of the protoconid. The metalophulid II is absent. The protoconid hind arm is merged with the ectolophid and it never protrudes in the mesosinusid (Fig. 1i, j). The mesolophid is usually present; only two specimens lack it (TGR-C/1). It is short and situated at the intersection of the posterior arm of the protoconid and the ectolophid (Fig. 1i, j). In one tooth (TGR-C/1), it is longer, curved and connected to the metaconid. The ectomesolophid is absent (Fig. 1i, j). The hypolophulid is short and wide; it is connected to the posterior part of the ectolophid. The hypoconid hind arm is always absent. The mesosinusid is closed by a small cingulum. The sinusid is narrow; it is retroverse in some specimens (TGR-C/2; TGW-A/2a). A cingular ridge connects the hypoconid with the protoconid at the labial border of the sinusid in 13 cases (TAR-A/2; TGR-C/1 (6); TGR-C/2 (3); TGW-A/2a (2); IKH-A/5).

**m3**: The labial anterolophid is long and reaches the protoconid. The lingual anterolophid is also long and connected to the metaconid (Fig. 1k, l). The metaconid displays a short posterior spur that develops towards the entoconid (Fig. 1l). The metalophulid I is present and connected to the anterolophulid or lingual anterolophid (Fig. 1k, l). The metalophulid II is absent. The protoconid hind arm is merged with the ectolophid. The mesolophid is sometimes present (TGR-C/2; TGR-C/1; DEL-B/12) and is always long and reaching the lingual border; in some cases, it is curved and connected to the hypolophulid or entoconid (Fig. 1k; DEL-B/12; TGR-C1; TGR-C/2). The entoconid is reduced and bears two spurs, one anterior and one posterior, both forming with the entoconid a characteristic “Y” shape (Fig. 1l). The ectomesolophid is always absent. The hypolophulid is connected to the point where the anterior part of the hypoconid and the ectolophid are connected (Fig. 1l). The hypoconid hind arm is absent. The mesosinusid is usually closed by a cingulum. The sinusid is short, narrow and transversal; it can be closed by a small cingulum (Fig. 1l).

**Remarks**: The most characteristic traits of the studied material are large size (Table 2), hypsodonty, rounded cusps, bunodont pattern and flat wear. Relative small anterocone, anterolophule joined to the lingual part of the anterocone and small spur on the anterocone on the M1. The upper molars have lingual cingula, oblique entoloph, metalophule connected to the posteroloph, and the shallow and weak protosinus. The m1s have no anterolophulid and both metalophulids, I and II, on the same specimen, as well as short mesolophids and ectomesolophids on the m1s but long mesolophid connected to the reduced entoconid on the m3. Some of these features are present on *Bagacricetodon tongi* (Gomes Rodrigues et al. 2012). The material from Mongolia differs from *B. tongi* by having posteroloph on the upper molars, longer metalophs on the M3s and shorter m1s. The above-mentioned characteristic traits of the Mongolian fossils fit with the description and the figures that Lopatin (2004) gives for the type locality and holotype (an M1) of *Aralocricetodon schokensis*, the only species described of this genus. That description differs only by the longer lingual anteroloph on the M2, longer mesoloph on the M3; the more-developed mesolophid and by the more-developed ectomesolophid on the m1. Lopatin (2004) stated that the hypolophulid on m1 adjoins the central region of the hypoconid. However, as visible on the illustrations of Lopatin 2004 (page S281, fig. 37h), the hypolophulid is connected to the ectolophid, as it is on the Mongolian material. Similarly, Lopatin (2004) stated that the m2 has no mesolophid and that the posterior arm of the protoconid is well developed. In our opinion, this is a misconception of the structure, and Lopatin (2004; fig. 37j) in fact illustrates a mesolophid on the m2.

We have compared the Mongolian material with the casts from level 2 at Altynschokysu stored at NHMW. Fossils from Kazakhstan and Mongolia share, besides the above-mentioned characters, a number of characteristics such as metalophule joined to the posteroloph on M2, absence of lingual anteroloph on M2 and M3, long mesoloph on M3, rounded and large anteroconid, lack of anterolophule, curved metalophulid II, short mesolophid and ectomesolophid on the m1 and long mesolophid connected to the reduced entoconid on the m3.

*Aralocricetodon* is present in the Valley of Lakes and Kazakhstan as well (Bendukidze 1993; Lopatin 2004; Bendukidze et al. 2009; Daxner-Höck et al. 2010; Maridet et al. 2014). This genus has been classified within Cricetodontinae sensu stricto (Bendukidze 1993; Lopatin 2004) and, also, as a member of the subfamily Tachyoryctoidinae within Muridae (Bendukidze et al. 2009). Recently, Wang and Qiu (2012) included *Aralocricetodon* in the family Cricetidae instead of Tachyoryctoididae. This is based on the general occlusal structures of the molar, and on that *Aralocricetodon* is much smaller than the members of Tachyoryctoididae (Wang and Qiu 2012). The most recent classification is proposed by Maridet and Ni (2013). They included *Aralocricetodon* in a cladistic analysis, and the results confirm its classification into the family Cricetidae but rather suggest an ascription to the subfamily Cricetopinae.

Genus *Bagacricetodon* Gomes Rodrigues et al., 2012

*Bagacricetodon tongi* Gomes Rodrigues et al., 2012

Fig. 2

**Fig. 2:**
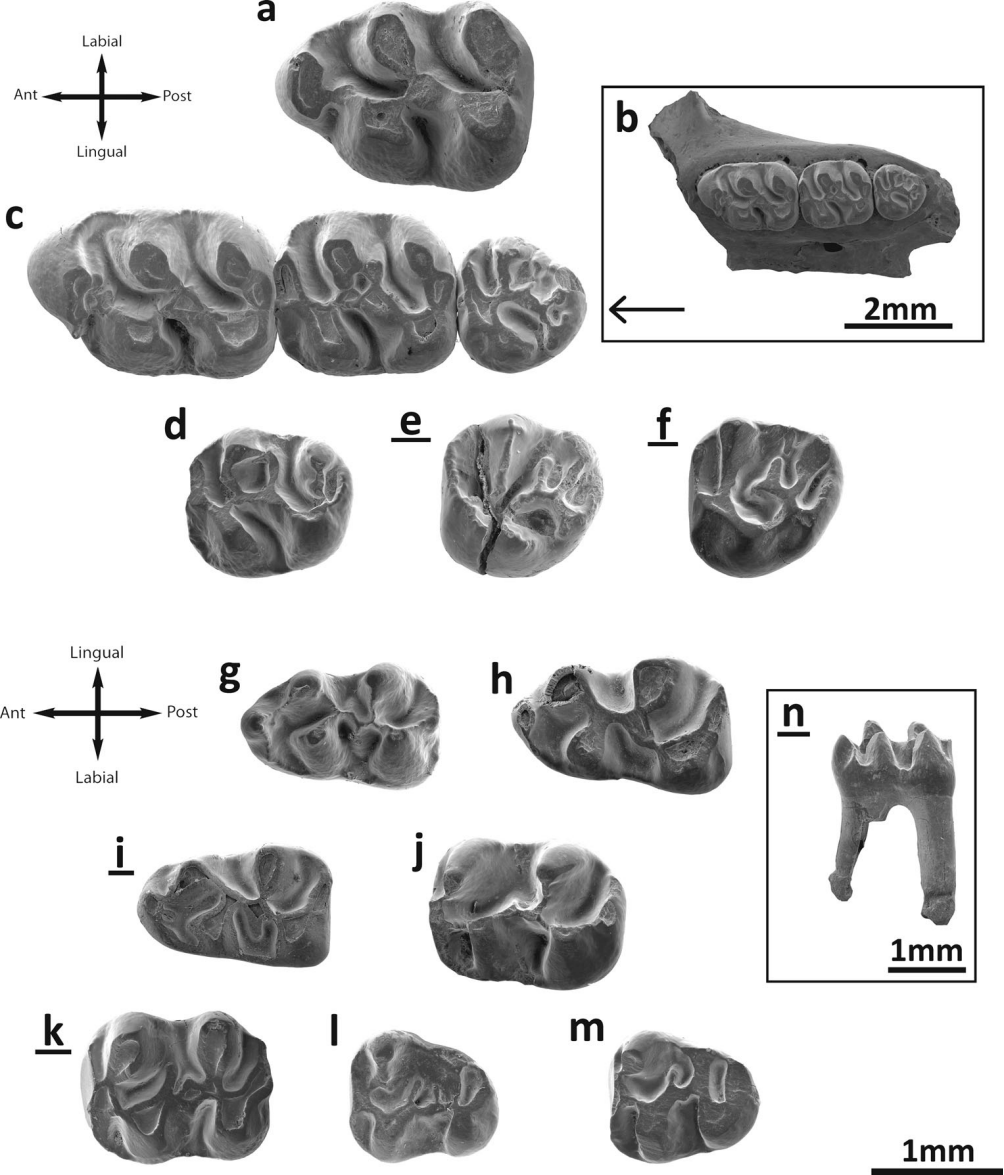
*Bagacricetodon tongi* and *B.* cf. *tongi* from the Valley of Lakes. **a** Taatsiin Gol Right locality, fossil layer TGR-C/1, left M1 (NHMW2015/0316/0001). **b** Toglorhoi locality, fossil layer TGW-A/2b, left maxilla (NHMW2015/0318/0004). **c** Left maxilla (NHMW2015/0318/0004). **d** Left M2 (NHMW2015/0318/0005). **e** Taatsiin Gol Right locality, fossil layer TGR-C/1, inverted right M3 (NHMW2015/0316/0002). **f** Toglorhoi locality, fossil layer TGW-A/2a, inverted right M3 (NHMW2015/0317/0002). **g** Fossil layer TGW-A/2b, left m1 (NHMW2015/0318/0009). **h** Left m1 (NHMW2015/0318/0007). **i** Taatsiin Gol Right locality, fossil layer TGR-C/1, inverted right m1 (NHMW2015/0316/0004). **j** Toglorhoi locality, fossil layer TGW-A/2b, left m2 (NHMW2015/0318/0017). **k** Inverted right m2 (NHMW2015/0318/0021). **l** Abzag Ovoo locality, fossil layer ABO-083, left m3 (NHMW2015/0319/0001). **m** Toglorhoi locality, fossil layer TGW-A/2b, inverted right m3 (NHMW2015/0318/0024). **n** Inverted right m1 (NHMW2015/0318/0010)

### Synonymy

2014 *Eucricetodon bagus*—Maridet et al. table 3. p. 264.

(Only for the locality TGR-A/2b *pro parte*)

2014 *Aralocricetodon* aff. *shockensis*—Maridet et al. table 3. p. 264. (Only for the locality TGR-C/1)

**Original type locality**: UTL4 (Ulan II), lower upper Oligocene, Ulantatal, Inner Mongolia (China).

**Stratigraphical range**: Upper Oligocene (Mongolia—upper Oligocene: local biozones C and C1), for details, see figs. 30–31 in Daxner-Höck et al. (2017) (this issue).

**Geographical Range**: Central Asia.

**Material**: see Table 1. Taatsiin Gol Right locality, fossil layer TGR-C/1; catalogue numbers (CN): NHMW2015/0316/00001-4. Toglorhoi locality, fossil layer TGW-A/2a; CN: NHMW2015/0317/0001-2; TGW-A/2b; CN: NHMW2015/0318/0001-24. Abzag Ovoo locality, fossil layer ABO-083; CN: NHMW2015/0319/0001-2. Del locality; CN: NHMW2015/0320/0001-3.

**Measurements**: given in Table 2.

### Description

**M1**: A shallow groove on the anterior wall divides the anterocone in two parts (Fig. 2c) but does not cover the entire height of the cusp. Thus, in specimens with strong wear, it is undivided (Fig. 2a). The labial anteroloph is well developed; it starts from the base of the anterocone and is joined to the middle part of the base of the paracone. The lingual anteroloph is weaker than the labial one (Fig. 2a) and does not reach the protocone. The anterocone bears a posteriorly directed spur in some specimens (Fig. 2c; TGR-C/1), but it is short. It can be connected to the anterolophule in some molars (Fig. 2c). The anterolophule is present and connected to the lingual lobe of the anterocone, or it can be double (Fig. 2c). The distal protolophule is strongly oblique, short (Fig. 2a, c) and joined to the entoloph. The entoloph is short and longitudinal; it is enlarged and forms a mesocone in most specimens. The protocone hind arm is merged with the entoloph. The protocone has a spur at its posterior wall. The entomesoloph is always absent. The metalophule is short, wide and connected to the posterior wall of the molar (Fig. 2a, c). The metacone is more or less fused with the posterior wall (Fig. 2a, c). The labial posteroloph is lacking. The anterosinus and mesosinus are long, curved and deep; they are closed by well-developed cingula. The sinus is proverse and closed by a small cingulum (Fig. 2c).

**M2**: The labial anteroloph is strong, wide and long; it is connected to the paracone. The lingual anteroloph is weaker but still well developed. The distal protolophule is present in all molars; it is short, wide and connected to the entoloph (Fig. 2b, c). The paracone spur is present in all specimens. In two molars (TGW-A/2b), it is joined to the mesoloph (Fig. 2c, d). The mesoloph is always present and well distinguished; its length is at least about half that of the mesosinus length or longer, almost reaching the labial border. The mesosinus is closed by a labial cingulum (Fig. 2b, c). The entomesoloph is absent. The metalophule is short and wide and positioned strongly posterior; it is connected to the posterior wall of the molar. The metacone is more or less fused with the posterior wall. There is no labial posteroloph. Anterosinus and mesosinus are long, curved and deep; they are closed by well-developed cingula. The sinus is strongly proverse, closed by a small cingulum.

**M3**: The labial anteroloph is present and long; the lingual is weak and, in one specimen, absent (TGR-C/1). The mesial protolophule is connected to the anterolophule. The paracone bears a small spur which is labially displaced, almost situated on the labial border (Fig. 2c). The entoloph is present and connected to the anterolophule (Fig. 2c); it can also be incomplete (Fig. 2e, f). The anterior arm of the protocone is connected to the anterolophule, and the posterior arm is freely in one specimen (TGW-A/2b), being connected to the neoentoloph in the other two (TGR-C/1). The anterior part of the entoloph is missing in one molar (TGW-A/2a). The mesoloph is always present and well developed. There is a spur of enamel on the mesosinus in two specimens (Fig. 2c, f). The posterior part of the entoloph is longitudinally oriented and it is connected to the metalophule (Fig. 2e). The neoentoloph is present in two out of three cases. The sinus is short and transversal when the neoentoloph is present, and long, anteriorly curved when the neoentoloph is missing. The hypocone is quite reduced. The metalophule is connected to the anterior arm of the hypocone. The posteroloph is always present but it is short and not connected to the metacone. The mesosinus is closed by a cingulum (Fig. 2e), by a style or can remain open.

**m1**: This molar has an elongated shape. The anteroconid is rounded and situated on the longitudinal axis of the occlusal surface. The labial anterolophid is a well-developed ridge that connects the anteroconid with the labial part of the protoconid. The lingual anterolophid and the anterolophulid are always absent. In one specimen (Fig. 2i), a small spur is present on the posterior side of the anteroconid, but it does not reach the protoconid. Both metalophulids are present. The metalophulid II has a very particular shape: it is oblique, straight and connected to the ectolophid. The metalophulid II, the ectolophid and the anterior arm of the hypocone have the same orientation and they draw a straight, oblique line (Fig. 2i). The posterior arm of the protoconid is joined to the intersection of the metalophulid II and ectolophid. The mesoconid is present and large (Fig. 2g–i). The mesolophid is not present; only one molar has an enlargement of the enamel that may be an incipient mesolophid (Fig. 2g). The same condition holds for the ectomesolophid, but it is well developed in two cases (Fig. 2g, i). The hypolophulid is short and connected to the mesoconid or to the posterior part of the ectolophid (Fig. 2g). The hypoconid hind arm is always absent. An external sulcus is visible on the posterior side of the hypoconid. The anterosinusid is weak but distinguishable. The mesosinusid is narrow and open (Fig. 2g). The sinusid is short, transversally directed and closed by a cingulum.

**m2**: Both labial and lingual anterolophids are present and equally well developed. The metalophulid I is present and connected to the labial anterolophid. The anterior arm of the protoconid is also connected to the lingual anterolophid. The point where the anterolophids are connected is enlarged and can be described as a small anteroconid. The metalophulid II is absent. The posterior arm of the protoconid is merged with the ectolophid in the material from two localities (TGW-A/2b; DEL-B/12). In other cases, the protoconid hind arm can extend to the metaconid (TGW-A/2b; Fig. 2k). The mesoconid is present and well developed (Fig. 2k), although it is not well distinguished on specimens without wear. The mesolophid is always present and short (Fig. 2j). The ectomesolophid is usually present (Fig. 2k) but short. Some specimens lack it (Fig. 2j). The hypolophulid is short, wide and connected to the posterior part of the mesoconid (Fig. 2k). The hypoconid hind arm is present in some molars. The posterior side of the hypoconid bears an external sulcus. The mesosinusid is wide and open. The sinusid is wide, transversal and closed by a small cingulum (which can be absent) (Fig. 2k).

**m3**: The labial anterolophid is long and reaches the protoconid. The lingual anterolophid is also long and connected to the metaconid. The metalophulid I is present and connected to the lingual anterolophid. The metalophulid II is absent, but one molar (Fig. 2l) bears a spur on metalophulid I, that could be interpreted as an incomplete distal metalophulid (although it is not connected to the ectolophid). The posterior arm of the protoconid is prolonged on the mesosinusid and ends freely. The ectolophid is long and thin; it bears a mesolophid in two cases out of total sample (Fig. 2l, m). The ectomesolophid is always absent. The entoconid is reduced and small. The hypolophulid is attached to the posterior part of the ectolophid. The hypoconid hind arm is absent. The mesosinusid is wide and closed by a cingulum. The sinusid is wide and open.

**Remarks**: The teeth of the studied assemblages are almost homogeneous in size and morphology. A size decrease in the younger localities is evident for all the elements except m3 (Table 2). The most characteristic features are the relative simple occlusal pattern, without extra folds; the tall crowns; the rounded cusps; the strongly oblique distal protolophule and metalophule on the upper molars as well as the presence of a metalophulid II and ectolophid on the lower molars. The absence of a posteroloph on M1 and the presence of a mesoconid on m1 and m2 are also particular traits that are commonly present. The Mongolian material also exhibits a distal protolophule joined to the entoloph and a weak posteroloph on M1; a short lingual anteroloph on M2; a metalophulid I on m1; and a small and reduced entoconid on m2. The presence of a large number of shared characters led us to assign the Mongolian material to *B. tongi*, found in Ulantatal (China). The Chinese material (Gomes Rodrigues et al. 2012) is, in average, slightly smaller than our material from Mongolia. However, the figured M2s illustrate a noticeable size variation (Fig. 2c, d). A similar important size variation has already been observed at the type locality of *B. tongi* as well (Ulantatal 6 M2 length—min 1.29, max 1.50; width—min 1.10, max 1.52). Consequently, both M2s from TGW-A/2b fall within the range size of the type population. Likewise, the M3s from TGR-C/1 and TGW-A/2a (Fig. 2e, f; Table 2) are characterised by their bigger size compared with the M3 from TGW-A/2b (Fig. 2c; Table 2). Only one M3 is known in the type material from Ulantatal, and the size (1.06 × 1.02) is closer to the latter M3. The morphology of the above-mentioned specimens is otherwise similar. We cannot evaluate the size variations based on such a small sample. Thus, it is so far not possible to know if this size variability illustrates a large range size of *B.* cf. *tongi* or if a second taxon, similar in morphology, is also present. We therefore tentatively refer all the M2s and M3s from TGR-C/1 and TGW-A/2a to *B.* cf. *tongi* until more material is available to evaluate the size range.

**Fig. 3:**
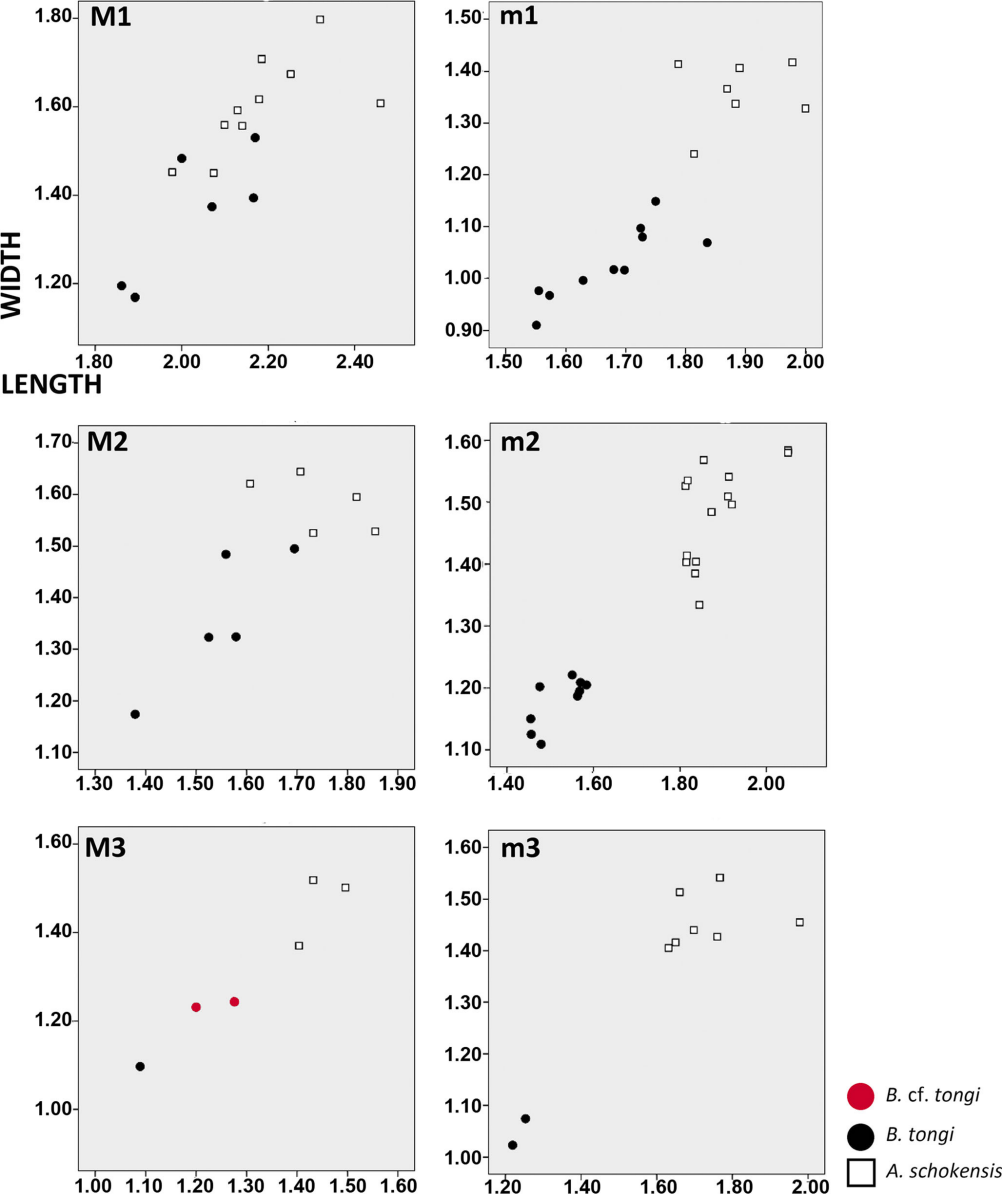
Length/width scatter-diagram of the cheek teeth of *Bagacricetodon tongi* and *Aralocricetodon schokensis* from the Valley of Lakes (Mongolia)

*B. tongi* has a similar but somewhat smaller size than *A. schokensis* (Fig. 3). Despite these close sizes, Gomes Rodrigues et al. (2012) did not include *Aralocricetodon* in the differential diagnosis of *Bagacricetodon*. In the frame of the present study, we compared our material with the casts of *A. schokensis* from level 2 at Altynschokysu stored at NHMW and with the original descriptions (Lopatin 2004). We found numerous differences supporting the validity of the genus. The anterocone of M1 in *A. schokensis* is undivided (Lopatin 2004; Fig. 1a, b), whereas it is divided in *B. tongi* (Fig. 2c). The posteroloph on the M1 is developed and conspicuous in *A. schokensis* (Fig. 1a, b) but absent in *B. tongi* (Fig. 2a, c). The distal protolophule of the M1 is slightly oblique and connects to the anterior part of the ectoloph in *A. schokensis* (Fig. 1a, b), whereas it is strongly oblique and connects directly to the mesocone in *B. tongi* (Fig. 2a, c). The mesoloph on M3 is clearly larger in *A. schokensis* (Fig. 1e, f) than in *B. tongi* (Fig. 2e, f). The m1 also differs in the two species: the posterior metalophulid is curved and connected to the protoconid in *A. schokensis* (Fig. 1g, h), whereas it is oblique, straight and connected to the ectolophid in *B. tongi* (Fig. 2g–i). The posterior arm of the protocone is connected to the ectolophid on m1 in *A. schokensis* (Fig. 1g, h), and it is joined to the intersection of the metalophulid II and ectolophid in *B. tongi* (Fig. 2g–i). Also, the ectomesolophid on *A. schokensis* is less developed (Fig. 1g, h) than in *B. tongi* (Fig. 2g, i). Moreover, the m1s of *A. schokensis* are bigger (Table 2) and have different proportions (*L*/*W* index 1.504, 1.341 and 1.362 versus 1.620 in *B. tongi*). The mesolophids are more developed on the m2s of *A. schokensis* (Fig. 1j) than in *B. tongi*, where they can be absent (Fig. 2j). The same holds true for the mesolophids on the m3s: they can reach the lingual border or join the hypolophulid in *A. schokensis* but are short in *B. tongi* (and end freely) (Fig. 2l, m). Nevertheless, *Bagacricetodon* and *Aralocricetodon* also share some common features such as the close size, the pear-shaped anterocone in M1, the oblique protocone and sinus in all upper molars, the rounded shape with a weakly developed hypocone in M3, the absence of anterolophulid and the well-developed anterior metalophulid in m1. These observations suggest that *Bagacricetodon* and *Aralocricetodon* might actually be closely related.

We also compared our material with the casts from level 2 at Altynschokysu stored at NHMW under the name *Eumyarion tremulus* but not figured in Bendukidze et al. (2009) (although we have already stated our doubts about the presence of *Eumyarion* in Asia; see López-Guerrero et al. 2016). Fossil teeth from Kazakhstan and Mongolia share a number of features such as strong oblique distal protolophule and metalophule, absence of paracone spur, small spur on the anterolophule and slightly split anterocone on M1, paracone spur on M2, presence of an ectomesolophid and mesoconid on the lower molars, metalophulid II strongly oblique and connected to the posterior arm of the protoconid and no anterolophulid on m1. Moreover, the teeth are similarly sized and have a long m1, reflected by their *L*/*W* ratio (Table 2). Studying the collection from Altynschokysu is beyond the scope of the present paper. Nonetheless, the similarities of the Kazakhstani specimens and *B. tongi* are remarkable. A revision of the molars from Altynschokysu is recommended to confirm or reject the presence of *B. tongi* at Altynschokysu, level 2. Its presence would imply that another shared taxon is present in these three regions apart from those already found (López-Guerrero et al. 2016, 2017, this issue).

*Eocricetodon* Wang, 2007

*Eocricetodon meridionalis* (Wang and Meng, 1986)

Fig. 4

**Fig. 4:**
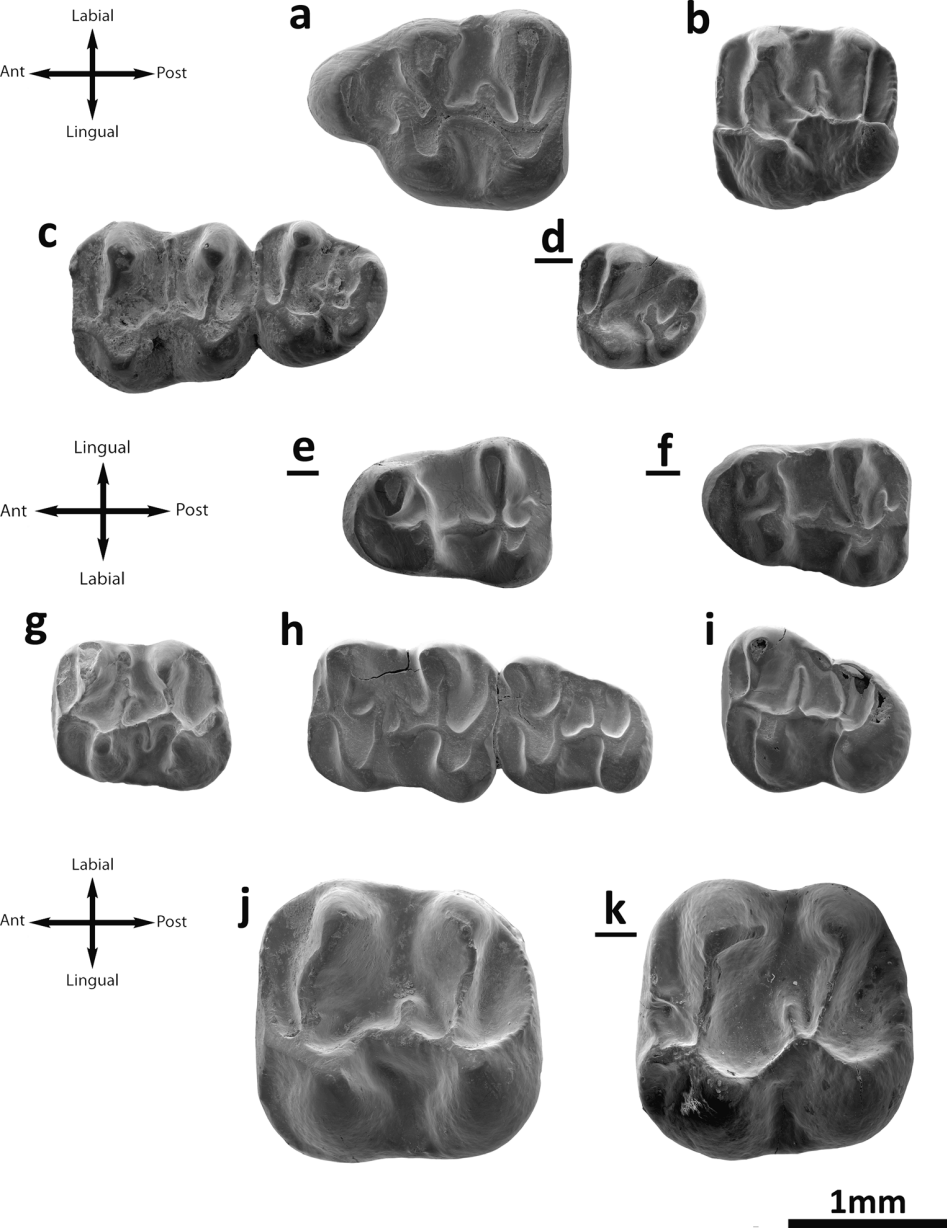
*Eocricetodon meridionalis* and *E.* cf. *meridionalis* from the Valley of Lakes. **a** Unkhektseg locality, fossil layer UNCH-A/3B, left M1 (NHMW2015/0311/0001). **b** Hsanda Gol locality, fossil layer SHG-C/1, left M1 (NHMW2015/0303/0001). **c** Del locality, fossil layer DEL-B/7, left M2-M3 (NHMW2015/0300/0001). **d** Avzag Ovoo locality, fossil layer ABO-A/3, inverted right M3 (NHMW2015/0299/0001). **e** Taatsiin Gol Right locality, fossil layer TGR-B/1, inverted right m1 (NHMW2015/0307/0001). **f** Toglorhoi locality, fossil layer TGW-A/2a, inverted right m1 (NHMW2015/0310/0005). **g** Taatsiin Gol Right locality, fossil layer TGR-ZO/2, left m2 (NHMW2015/0309/0002). **h** Left m2-m3 (NHMW2015/0310/0002). **i** Taatsiin Gol Right locality, fossil layer TGR-AB/22, left m3 (NHMW2015/0306/0003). *Witenia* sp. **j**, Unkhektseg locality, fossil layer UNCH-A/3B, left M2 (NHMW2015/0537/0001). *Paracricetodon* sp. **k**, Taatsiin Gol Right locality, fossil layer TGR-A/14, inverted right M2 (NHMW2015/0533/0001). All molars are at the same scale

### Synonymy

2014 *Eucricetodon* aff. *caducus*—Maridet et al. table 3. p.

264. (Only for DEL-B/12 and TAT-E/27

2014 *Eucricetodon* aff. *bagus*—Maridet et al. table 3. p. 264.

(Only for IKH-A/1)

2014 *Eucricetodon* sp. 1—Maridet et al. table 3. p. 264

2014 *Eocricetodon* cf. *meridionalis*—Maridet et al. table 3.

p. 264. (Only for IKH-A/3-4 *pro parte*)

**Original type locality**: Caijiaching locality, Yunnan Basin, lower Oligocene, China.

**New localities**: see Table 1.

**Stratigraphical range**: Upper Eocene–upper Oligocene. (In Mongolia—Oligocene: local biozones B, C and C1).

**Geographical range**: Central Asia.

**Material**: see Table 1. Abzag Ovoo locality, fossil layer ABO-A/3; catalogue numbers (CN): NHMW2015/0299/0001. Del locality; CN: NHMW/2015/0300/0001-3. Ikh Argalatyn Nuruu locality, fossil layer IKH-A/1; CN: NHMW/2015/0301/0001. Hsanda Gol locality, fossil layer SHG-AB/17-18; CN: NHMW/2015/0302/0001-2; SHG-C/1 NHMW/2015/0303/0001-2. Tatal Gol locality, fossil layer TAT-E/27; CN: NHMW/2015/0304/0001. Taatsiin Gol Right locality, fossil layer TGR-A/13; CN: NHMW/2015/0305/0001; TGR-AB/22; CN: NHMW/2015/0306/0001-3; TGR-B/1; CN: NHMW/2015/0307/0001-3. TGR-C/5; CN: NHMW/2015/0308/0001; TGR-ZO/2; CN: NHMW/2015/0309/0001-4. Toglorhoi locality, fossil layer TGW-A/2a; CN: NHMW/2015/0310/0001-7. Unkheltseg locality, fossil layer UNCH-A/3; CN: NHMW/2015/0311/0001.

**Measurements**: given in Table 2.

### Description

**M1**: The valleys are wide and shallow. The labial cusps are transversally elongated and low. The anterocone is labial-lingually elongated; it is undivided and displaced labially (Fig. 4a). Both lingual and labial anterolophs are well developed. They are attached to the protocone and paracone, respectively. The anterolophule is absent. The anterior arm of the protocone ends freely on the anterosinus and is wide (Fig. 4a). The distal protolophule is short and joined to the posterior arm of the protocone. The posterior spur of the paracone is present but extremely short and directed towards the edge of the mesosinus. The tooth has a short and wide mesoloph (Fig. 4a). The metalophule is transversal and connected to the hypocone (Fig. 4a). The labial posteroloph is long. The mesosinus is closed by a style. The sinus is slightly proverse and closed by a small cingulum.

**M2**: Both the lingual and labial anterolophs are well developed, although the latter is longer than the former. The anterolophule is a well-distinguished crest. The distal and mesial protolophules are present. The mesial protolophule is always present (Fig. 4b, c) and slender; it is connected to the intersection of the anterolophule and the anterior arm of the protocone. The distal protolophule is present but weaker than the mesial one; it is incomplete in one case out of two (Fig. 4c). In one case, both protolophules are present (TGW-A/2a). The protolophule spur and paracone spur are absent. The entoloph is longitudinally oriented and lingually curved anteriorly. The posterior arm of the protocone is connected to the entoloph. The two specimens display a mesoloph that is long, in one case reaching the labial border (Fig. 4c). The entomesoloph is absent. The metalophule is connected to the entoloph, clearly before the hypocone in one specimen (Fig. 4b). The posteroloph is long, well developed and connected to the metacone. The anterosinus is wide and long. The mesosinus is closed by a style in one case (Fig. 4b). The sinus is always proverse and open.

**M3**: The labial anteroloph is present and long, and the lingual is absent. The mesial protolophule is present and connected to the short anterolophule. The paracone spur is absent. The entoloph is an isolated ridge in the middle of the tooth. Both anterior and posterior parts of the entoloph are incomplete (Fig. 4c, d). A long mesolophid is present in the middle of the entoloph. The posterior arm of the protocone is connected to the anterior arm of the hypocone in one molar (Fig. 4c), and this posterior arm is also connected to the entoloph. All these connections among the ridges draw a cross in the middle part of the tooth. On the other specimen (Fig. 4d), the anterior part of the entoloph is absent. Hypocone is not reduced but is posteriorly displaced. The metalophule is connected to the hypocone. The posteroloph is absent in one molar (Fig. 4c) and present but short in the other (Fig. 4d). The mesosinus is open. The sinus is short and retroverse.

**m1**: This molar is elongated. The valleys are wide and shallow and the cusps are low. The anteroconid is transversally elongated, situated on the longitudinal axis of the labial anterolophid. In some specimens (Fig. 4f), it is so elongated that is barely distinguished. The labial anterolophid is a well-developed ridge that connects the anteroconid with the labial part of the protoconid. The lingual anterolophid is also present and connects the anteroconid with the metaconid. The anterolophulid is usually absent (Fig. 4e, f) but in some cases (TGR-B/1; TGR-AB/22) it is present and connected to the middle part of the anteroconid. The metalophulid I is present in some specimens (Fig. 4f; TGR-AB/22) but is incomplete. The metalophulid II is always present, curved and connected to the posterior arm of the protoconid (Fig. 4e, f). The ectolophid is longitudinally straight and labially displaced (Fig. 4e, f); it is joined to the posterior part of the protoconid. The mesolophid is weak (Fig. 4f) or absent; it is long in one specimen out of the total sample (15). The ectomesolophid is absent in all molars but one (TGR-AB/22). The entoconid spur is present in one molar (TGR-AB/22). The hypolophulid is short and connected to the anterior arm of the hypoconid. The hypoconid hind arm is present in four cases (Fig. 3f; TGR-AB/22; TGW-A/2a; IKH-A/1). The mesosinusid is wide and closed by a cingulum. The sinusid is short and wide, transversal and either open or closed by a small cingulum (Fig. 4e).

**m2**: The labial anterolophid is long, thin and reaches the protoconid. The lingual anterolophid is weakly developed (Fig. 4h) or equally developed as the labial one (Fig. 4g). The metalophulid I is present and connected to the anterolophulid. The metalophulid II is absent. The protoconid hind arm is long and ends freely in the mesosinusid (Fig. 4g); it can be connected to the ectolophid (Fig. 4g). The ectolophid is oblique, connected to the protoconid and bears a mesolophid that can be long. The ectomesolophid can be present (Fig. 4g) or absent (Fig. 4h). The hypolophulid is connected to the point where the ectolophid and the anterior arm of the hypoconid meet. The hypoconid hind arm is absent. The posterolophid is long and an external sulcus is present on the posterior side of the hypoconid (Fig. 4h). The mesostylid is present in one specimen (Fig. 4g). The mesosinusid is closed by a cingulum (TGW-A/2a) or open (Fig. 4h). The sinusid is wide, transversal and short; it is closed by a small cingulum in one case (DEL-B/7) or open (Fig. 4h).

**m3**: Both labial and lingual anterolophids are present. In three cases (TGR-ZO/2; TGW-A/2a), the labial one is longer than the lingual one; in the other one (Fig. 4i), they are equally developed. The metalophulid I is present and connected to the anterior arm of the protoconid. The metalophulid spur is absent. The metalophulid II is always absent. The posterior arm of the protoconid is well developed (Fig. 4i) and reaches the lingual border in one specimen (Fig. 4h). The ectolophid is longitudinally oriented (Fig. 4i) or oblique (Fig. 4h). The mesolophid is not present in the remaining teeth (Fig. 4i). The ectomesolophid is missing. The entoconid is small and reduced (Fig. 4i). Hypolophulid connected to the intersection of the ectolophid and the anterior arm of the hypoconid. The hypoconid hind arm is absent. The mesosinusid is closed by a stylid (Fig. 4i) or by a cingulum. The sinusid is transversal, wide and open.

**Remarks**: The Mongolian material studied herein presents a relatively simple occlusal pattern. Among the main characters displayed are the brachyodont molars with wide valleys and thick crests, the transversal metalophule on the upper molars, the elongated anterocone with a distinguished prelobe on M1, the long mesoloph on M2 and M3 and the absence of the anterior part of the entoloph on M3. This material also presents a straight and labially displaced ectoloph on the lower molars and a transversally elongated anteroconid with both anterolophs and absent anterolophule on the m1. Another important trait is the long posterior arm of the protoconid on m2 and m3. Most of these features are included in the diagnosis of *Eocricetodon meridionalis* (Wang and Meng, 1986). Wang and Meng (1986) also described a mesostyle on M1 and a posterior arm of the protoconid longer than the mesolophid. These traits are also present in the Mongolian fossils.

*E. meridionalis* was firstly described as a member of *Eucricetodon*. Wang (2007) created *Eocricetodon* to gather the small-sized cricetids with primitive characters. Another species of *Eocricetodon* was described in Inner Mongolia. *Eocricetodon borealis* Wang, 2007 was recovered at the Railways Station of Erenhot (Houldjin Fm) and dated as Late Eocene. *E. borealis* is also a small cricetid, but the length of its M1 is longer than the length of the M1 from Mongolia (1.87 vs 1.60 mm). In addition, *E. borealis* exhibits a more pronounced and more slender anterior lobe on M1 (Wang 2007) than *E. meridionalis*. Moreover, the anterior arm of the protocone on M1 is longer and reaches the anterocone at its labial part.

*Eucricetodon occasionalis* Lopatin, 1996 has a similar size and morphology (López-Guerrero et al. 2017, this issue). However, *E. meridionalis* differs from *E. occasionalis* by its mesostylid and entoconid spur on M1, its transversal metalophule, its lingual anterolophid, its constriction on the posterolophid and the absence of a hypoconid hind arm on m2, its well-developed posterior arm of the protoconid on m2 and m3 that reaches the lingual border, its longitudinally oriented ectolophid on m3, its reduced entoconid on m3 and its open sinusid on m3. Among the studied material, the m3s from TGR-ZO/2 and TGW-A/2a are smaller than the type material (Table 2; Wang and Meng 1986). However, they are not morphologically different. They share, among others, the long posterior arm of the protoconid, the hypolophulid connected to the ectolophid and the reduced entoconid (Fig. 3h; Wang and Meng 1986). The type sample contains only two measurable m3s and the size variability cannot be assessed. Therefore, until the size ranges of *E. meridionalis* are better known, we assign these two m3s to *E.* cf. *meridionalis*.

*E. meridionalis* has been described by different authors as either a primitive taxon or derived species. Gomes Rodrigues et al. (2009) studied the collection from the Chinese Yunnan Basin (late Eocene) and recognised certain plesiomorphic traits in comparison with members of the Eucricetodontinae such as the complete anterolophule. Maridet and Ni (2013) performed a cladistic analysis including *E. meridionalis*, also from the Chinese late Eocene. Their results placed *Eocricetodon* species in a politomy with the clades Cricetopinae and Pseudocricetodontinae, which present more derived traits than genera such as *Eucricetodon*, *Atavocricetodon* or *Pappocricetodon* (Maridet and Ni 2013). This is congruent with Wang (2007), who stated that *Eocricetodon* exhibits more advanced traits than *Pappocricetodon* (late Eocene) and *Atavocricetodon* (early Oligocene) such as the metalophule attached to the hypocone. The morphology and age of our Mongolian material of *Eocricetodon meridionalis* also support the hypothesis of its derived pattern as suggested by Wang (2007) and Maridet and Ni (2013). Our material lacks an anterolophule or a spur on the anterocone (a basal trait according to Li et al. 2016); it is dated as early and late Oligocene, which would indicate the more derived pattern of the Mongolian material.

*Pappocricetodontinae* Tong, 1997

Genus *Witenia* de Bruijn et al., 2003

*Witenia* sp.

Fig. 4j

### Synonymy

2014 *Eucricetodon* aff. *asiaticus*—Maridet et al. table 3. p.

264. (UNCH-A/3B *pro parte*)

**Stratigraphical range**: Eo/Oligocene boundary-Early Oligocene (in Mongolia—early Oligocene: local biozone B).

**Geographical range**: Asia Minor and Central Asia.

**Material**: right M2 (NHMW2015/0537/0001) from Unkheltseg locality, UNCH-A/3 fossil layer.

**Measurements**: 2.01 × 1.94.

### Description

**M2**: Both lingual and labial anterolophs are well developed, but the lingual is slightly shorter. The mesial protolophule is present and connected to the anterior arm of the protocone. The posterior arm of the protocone is connected to the entoloph. The mesocone is absent. The entoloph is straight, thick and bears a short mesoloph. The paracone spur is absent. The metalophule is connected to the anterior arm of the hypocone. The posteroloph is long and has no constriction. The mesosinus is deep, narrow and open. The sinus is narrow, long and proverse; it is open.

**Remarks**: The size of the molars is similar to some species of *Paracricetodon*, but it lacks its characteristic retroverse sinus. The genus *Witenia* displays a similar size; *Witenia flava* de Bruijn et al., 2003 is smaller than the Mongolian material. The original diagnosis of *W. flava* remarks that the M2 possesses a double metalophule (de Bruijn et al. 2003), but it is simple and anteriorly directed in our molar. Note, however, that in the description and plates of *Witenia fusca* de Bruijn et al., 2003, the metalophule is simple and anterior. We found the same condition in *Witenia yolua* Gomes Rodrigues et al., 2012: it has a simple anterior metalophule. We therefore conclude that our specimen belongs to *Witenia*, but as this is based on only one molar, we cannot make a further specific assignation.

Genus *Paracricetodon* Schaub, 1925

*Paracricetodon* sp.

Fig. 4k

### Synonymy

2014 cf. *Erdinella* sp.—Maridet et al. table 3. p. 264.

**Stratigraphical range**: Early Oligocene (local biozone A).

**Geographical range**: Europe; Asia Minor and Central Asia.

**Material**: left M2 (NHMW2015/0533/0001) from Taatsiin Gol Right locality, TGR-A/14 fossil layer.

**Measurements**: 1.89 × 1.90 mm.

### Description

**M2**: Both anterolophs are present, but the lingual one is shorter than the labial one. The anterolophule is well distinguished. The strong anterior arm of the protocone connects to the anterolophule. The anterior arm of the protocone and the short mesial curving protolophule usually enclose a pit. A short mesoloph is present close to the metalophule. The entoloph is absent, but the protocone distal arm is well developed and connects the protocone with the hypocone; it is strongly oblique and straight. The distal protolophule and metalophule are parallel. The posterior spur of the paracone is present but short. The anterior arm of the hypocone is parallel to the anterior arm of the protocone. The transverse or slightly forward directed metalophule connects the metacone to the antero-labial side of the hypocone. The mesosinus is open and the sinus is transversal or even slightly retroverse and open.

**Remarks**: According to the emended diagnosis proposed by de Bruijn et al. (2003), the anterior arms of the protocone and hypocone on M1 and M2, in *Paracricetodon*, are well developed; the sinus of M1 and M2 is posteriorly directed. The latter trait is a very particular character which is rarely observed in other cricetids. Consequently, we assign the molar here studied to *Paracricetodon*. This genus has been found in some Oligocene localities from Europe and Asia Minor. Among the species of *Paracricetodon*, the Mongolian M2 has a size similar to *Paracricetodon kavakderensis* Ünay, 1989 and to *Paracricetodon kodjayarmensis* Ünay, 1989. These species, however, have a distal protolophule and a cingulum on the mesosinus. Given the scarcity of material, we prefer not to propose a new species but state that the combination of size and morphology of the Mongolian *Paracricetodon* seems to be exclusive for the Valley of Lakes.

## Remarks on palaeoecology

Taking the present study into consideration, all the cricetids from the Oligocene of Mongolia have been characterised (Daxner-Höck 2000; Daxner-Höck et al. 2010; López-Guerrero et al. 2015, 2016, 2017, this issue). This enables us to illustrate the main morphological transformations observed in Central Asia.

Dentally, the cricetid rodents from the Valley of Lakes exhibit a reduction and simplification of the occlusal pattern towards the end of the Oligocene. In biozones A and B, the cricetid faunas are dominated by species of *Eucricetodon* (López-Guerrero et al. 2017, this issue) and *Eocricetodon*. These species have complicated morphological patterns that involve a number of crests and ridges, and they also have a transversal orientation of the metalophule on the upper molars. This suggests that these animals had a mainly herbivorous diet (Evans et al. 2007). The presence of *Eucricetodon*, *Paracricetodon* and *Witenia* also in Europe suggests a preferential faunal interchange between Europe, Asia Minor and Central Asia at the beginning of the Oligocene.

In biozones C and C1, the morphology of the dental pattern of the species is simpler. *Aralocricetodon*, *Argyromys* and *Bagacricetodon* have less-developed secondary ridges such as mesoloph/ids, paracone spur or anterlophulid on the lower molars. Also, the orientation of the ridges such as the metalophule and metalophulid II is more oblique. The teeth of *Aralocricetodon* and *Argyromys* have a flat wear and, in general, are larger than those of the cricetids from biozones A and B. The species from the late Oligocene are more hypsodont than those from the early Oligocene. This suggests a diet change that introduces a faunal component (Evans et al. 2007) and a fossorial life style. These changes in the taxonomic composition of the cricetid faunas and in their dental patterns suggest an increasing aridification, successive loss of soft plants and opening of environments. This coincides with the global Oligocene Glacial Maximum (Zachos et al. 2001). Remarkably, the genera in biozones C and C1 are typical from other regions of Central Asia (Kazakhstan and China), and none are present in Europe or Asia Minor localities.

## Conclusions

Both the morphological and metrical features of the studied fossils led us to identify five genera: *Aralocricetodon*, *Bagacricetodon*, *Eocricetodon*, *Witenia* and *Paracricetodon*. These genera are found in sediments dated as early Oligocene up to early-late Oligocene (biozones A to lower part of C1). The present work completes the information previously published on the Valley of Lakes. The study of *Aralocricetodon schokensis* confirms its occurrence in Mongolia in biozones C and C1. *Bagacricetodon tongi* was known only from one locality in biozone C1 and has now been recovered in another five from biozone C. The presence of *Eocricetodon meridionalis* is confirmed and its stratigraphical distribution is extended to biozones C and C1. The present study describes the genera *Witenia* and *Paracricetodon* in Mongolia for the first time. The implications of finding these European and Asia Minor genera in Mongolia are very interesting and must be taken into consideration in further studies.

The species studied here present simple dental patterns, medium to large size, relatively high crowns and, in some cases, flat wear. This suggests that they incorporated faunal elements in their diets (simple morphology) along with the consumption of abrasive and fibrous plants (high crowns). Finally, the flat wear of *Aralocricetodon*, among other traits, suggests an adaptation to a fossorial life style.

The present work has increased our knowledge about the species of Cricetidae during a very interesting interval at the beginning of their evolutionary history, as well as to precisely identify the stratigraphical and geographical distribution of the studied genera. Nevertheless, some aspects about the systematics and phylogeny of certain taxa such as *Aralocricetodon* remain to be solved. Indeed, future cladistic analyses including all the Mongolian, Chinese and Kazakhstani species should help us to more precisely understand the phylogenetic relationships and evolution of this group.
